# A new role for proteins subunits of RNase P: stabilization of the telomerase holoenzyme

**DOI:** 10.15698/mic2020.09.730

**Published:** 2020-06-17

**Authors:** P. Daniela Garcia, Virginia A. Zakian

**Affiliations:** 1 Molecular Biosciences, The University of Texas at Austin, Austin, TX, 78712 USA.; 2 Lewis-Sigler Institute, Princeton University, Princeton NJ 08544-1014 USA.

**Keywords:** telomerase, telomere, RNase P, RNase MRP, telomerase RNA

## Abstract

RNase P, an RNA-protein complex, is essential for processing tRNAs. Three of the ten protein subunits of *Saccharomyces cerevisiae* RNase P (and a related complex, RNase MRP) co-purify with yeast telomerase, another RNA-protein complex. The three telomerase-associated proteins, Pop1, 6 and 7, bind to TLC1, the RNA subunit of telomerase. In a recent study (*Garcia et al.* Nat Commun), we used temperature sensitive alleles of the essential *POP* genes to determine their role in telomerase biogenesis. At permissive temperature, *pop* mutant cells grow normally, and the abundance of most proteins, including protein subunits of telomerase, is similar to wild type (WT). However, telomeres are short, and the amount of the mature telomerase holoenzyme is low. Unlike the RNA subunit of RNase MRP, TLC1 is more abundant in *pop* cells and properly folded, except at the Cs2a/TeSS domain where the Pop proteins bind. These defects correlate with defective movement of TLC1 from the cytoplasm, where it associates with telomerase proteins, back to the nucleus where it lengthens telomeres. Thus, Pop proteins are needed for the stable association of telomerase proteins with TLC1, and their reduction sequesters mature telomerase in the cytoplasm, away from its nuclear substrates.

RNase P is a ubiquitous RNA-protein complex that is essential for the processing of the 5' ends of tRNAs. *Escherichia coli* RNase P consists of a 350 nt RNA and a single protein. The RNA subunit is sufficient for catalysis *in vitro*. Eukaryotic RNase P's have a similarly sized RNA subunit (369 nt), but an expanded number of proteins (10), and their catalytic activity is protein-dependent (sizes and protein number are for *S. cerevisiae*) (**Fig. 1a**). A few non-tRNA RNAs are also processed by both bacterial and eukaryotic RNase P.

**Figure 1:**
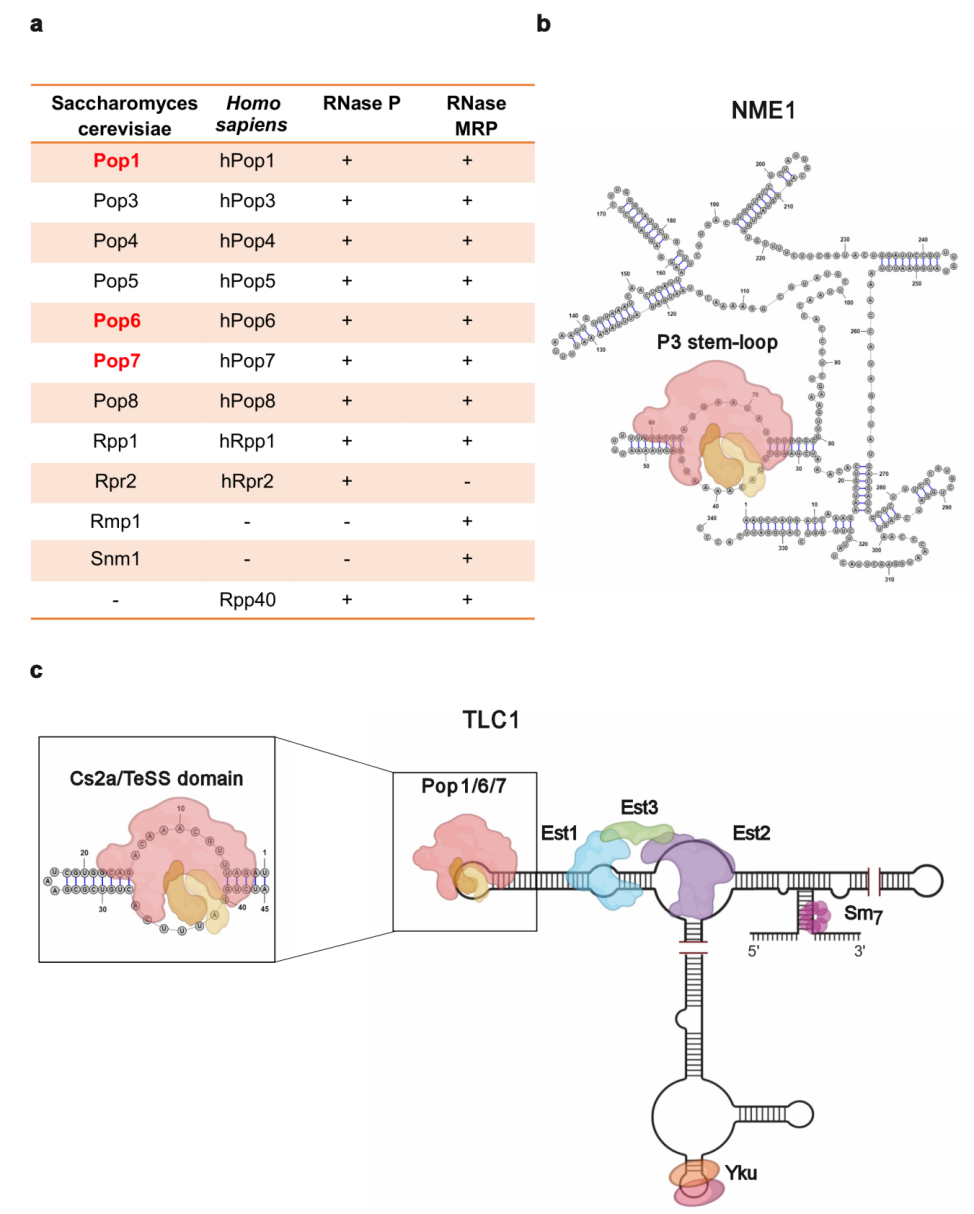
FIGURE 1. **(a)** Table presents names of proteins in RNase P and MRP in both *S. cerevisiae* and *H. sapiens;* (+) and (-) indicate presence or absence of the protein in the indicated RNase complex. Names of three Pop proteins that are telomerase-associated in *S. cerevisiae* are in red. **(b)** Structure of the 340 nt RNase MRP RNA subunit, NME, bound by Pop1 (red; ~100kDa), Pop6 (orange; ~18 kDa), Pop7 (tan; ~16 kDa) at the P3 stem-loop domain. **(c)** Diagram of the 1157 nt *S. cerevisiae* telomerase RNA, TLC1, bound by the core holoenzyme protein components. Expanded area depicts the Cs2a/TeSS (P3-like) domain in TLC1 to which Pop1, 6, and 7 bind. Images were made in BioRender (biorender.com).

RNase MRP, which processes ribosomal RNA and one mRNA, is a eukaryotic-limited RNA-protein complex. Although the RNA components of RNase P and MRP are different, all but a few of the protein subunits are present in both complexes. The two RNA subunits are mostly unrelated in sequence and structure, but each contains a P3 region of almost identical sequence and secondary structure. In budding yeast, a heterodimer of two subunits, Pop6 and Pop7, binds P3, and this complex recruits the larger Pop1 protein (**Fig. 1b**). Other protein subunits are recruited to the two RNAs via interactions with Pop1. Thus, Pop1/6/7 are the organizing core for both RNase P and MRP. All of the RNase P/MRP subunits in *S. cerevisiae* are encoded by essential genes, and most of the yeast proteins have human homologues (**Fig. 1a**).

Like RNase P/MRP, telomerase is an RNA-multi-protein complex that maintains the ends of chromosomes (telomeres) in most eukaryotes (**Fig. 1c**). Telomerase RNAs vary in size and sequence, but in all organisms, they are highly structured. In addition, all telomerase RNAs contain a short stretch whose sequence is complementary to the G-rich strand of telomeric DNA. This region base pairs to and provides a template for extension of the G-rich strand. The catalytic subunit of all telomerases, called Est2 in *S. cerevisiae,* are reverse transcriptases. Additionally, *S. cerevisiae* telomerase contains Est1 and Est3 that are also essential for telomerase action *in vivo*. Est1 promotes binding of the telomerase holoenzyme to telomeres, but its essential role is activating telomerase, perhaps by recruiting Est3 into the holoenzyme. Est1 is the only telomerase subunit whose abundance is cell cycle regulated, peaking in late S/G2 phase, the time of telomerase action.

In a 2015 paper, we used mass spectrometry to identify proteins that co-purify with *S. cerevisiae* telomerase. Pop1, 6, and 7, subunits of both RNase P and MRP, are all high confidence telomerase-associated proteins. The three Pop proteins are associated with both active (late S/G2) and inactive (G1 phase) telomerase. Their association is DNase-resistant. Thus, the three Pop proteins behave like integral subunits of telomerase. In contrast, none of the other RNase P/MRP proteins has significant telomerase association.

A 2016 paper from the Wellinger and Krasilnikov labs confirmed our finding that Pop1, 6 and 7 are telomerase associated. They demonstrated that the three Pop proteins bind to the Cs2a/TeSS domain, a P3-like region, in TLC1 RNA, which is near the Est1 binding site on the RNA (**Fig. 1c**). When the P3-like region is deleted, Est1 binding to the mutant TLC1 is low when measured by RNA immuno-precipitation (RNA-IP).

Meanwhile, we were carrying out experiments to determine if Pop proteins affect telomerase assembly and/or activity *in vivo*. Because *POP* genes are essential, we used temperature sensitive alleles of *pop1* and *pop6*. All experiments were carried out at 24°C (permissive temperature). At this temperature, *pop1* and *pop6* cells grow at near WT rates, and most proteins, including Est1 and Est2 are present at near WT amounts. However, levels of the mutant Pop proteins are significantly reduced. Many experiments were also done at 30°C, a semi-permissive temperature, where *pop* cells grow slowly but indefinitely, and protein levels are reduced, including Est1 (and to a lesser extent Est2). We reasoned that telomere phenotypes in *pop* cells at 24°C (especially if they are exacerbated at 30°C) are likely direct effects of Pop proteins on telomerase. All the mutant phenotypes we detect are suppressed by introducing a WT copy of the mutant *POP* gene.

Telomeres in *pop* cells are significantly shorter than WT telomeres, even at 24°C and shorter still at 30°C (~70% and ~60% of WT, respectively). This short telomere phenotype is not due to reduced Est1 or Est2. As the abundance of NME1, the RNA subunit of RNase MRP, is reduced in *pop* cells (~50 and 25% of WT at 24° and 30°C), we predicted that TLC1 would also be present in low amounts. However, TLC1 is actually more abundant in *pop* cells at both 24°C and 30°C, ~2 and 6x higher than in WT cells. The increased abundance of TLC1 in Pop-limited cells was surprising.

Given the well documented role of RNase P/MRP in RNA processing, we hypothesized that *pop* cells might be defective in TLC1 processing, and this defect might somehow lead to higher levels of TLC1. However, post-transcriptional changes at both the 5' and 3' ends of TLC1 occur normally in *pop* cells. Thus, Pop proteins do not affect processing of TLC1.

Next, using RNA-IP, we tested if defect(s) in TLC1 prevented its binding to its associated proteins. By RNA-IP, Est1 binding (and, to a lesser extent, Est2) to WT TLC1 is highly compromised in *pop* cells, even at 24°C (~14% of WT). This low binding is not due to reduced Est1 or 2. Binding of mutant Pop proteins to TLC1 is also low at 24°C, but in this case, lower binding can be explained by reduced Pop proteins. Likewise, the telomere association of the telomerase holoenzyme is reduced in both mutants at 24°C. The defects in telomerase assembly and its lower binding to telomeres likely explain the short telomere phenotype of *pop* cells.

Previous *in vitro* data suggest that the complex secondary structure of NME1 is Pop-protein dependent. If Pop protein deficiency results in improper folding of TLC1, this defect could explain its impaired binding to telomerase proteins. To test this idea, we used DMS-MaPseq, a method developed by Sylvia Rouskin's lab (MIT), for high through put analysis of RNA secondary structures. We determined the structure of both NME1 and TLC1 RNAs in *pop* and WT cells at both 24 and 30°C in DMS treated and untreated cells. As anticipated from earlier work, the secondary structure of NME1 was globally perturbed in *pop* cells even at 24°C. Strikingly, seven nucleotides in the P3-like region, where Pop6 and Pop7 bind, is the only portion of TLC1 that is altered in 24°C grown *pop* cells.

The altered DMS accessibility of the P3-like domain was expected as levels of Pop proteins are reduced at 24°C in *pop* mutant cells. However, we were surprised that the structures of Est1, Est2, Yku, and the Sm_7_ ring binding sites are indistinguishable in *pop* and WT cells, as by RNA-IP, Est1 (and to a lesser extent) Est2 binding to TLC1 is low at 24°C. One possibility is that our methods are not sensitive enough to detect structural differences at these sites. However, the structure of NME1 RNA in the same cells is globally perturbed at 24°C. Also, the Est1 binding site on TLC1 is significantly altered at 30°C in *pop* cells, consistent with less Est1 in *pop* cells at this temperature. The data are also sensitive enough to detect greater DMS-sensitivity in the P3-like domain in *pop6* compared to *pop1* cells, consistent with the Pop6-dependence of Pop1 binding to this region. The significance of the differences we detect are robust due to the large number of reads (680,000 per nt) and biological triplicates of DMS treated and untreated controls for each strain. Finally, the statistical methods used by our collaborator Sharon Aviran (UC Davis) can detect and assess the importance of structural changes, even if they are present in only a subset of molecules.

The DMS-MaPseq method monitors the secondary structure of RNA *in vivo*. The WT structure of the Est1, Est2, Yku and Sm_7_ binding sites in *pop* cells seen by this method suggest that these binding sites are occupied to a similar extent in *pop* and WT cells. In contrast, RNA-IP is measured in cell extracts and does not use cross-linkers to preserve RNA-protein interactions. Thus, WT-like occupancy of these binding sites in *pop* cells, as measured by DMS-MaPseq, and their low occupancy as measured by RNA-IP suggest that Est1 (and to a lesser extent Est2) bind much less stably to TLC1 in *pop* compared to WT cells. Preliminary RNA-IP data suggest that Yku binding to TLC1 is also impaired. We suggest that Pop proteins are required for higher order folding of TLC1, and this folding stabilizes the binding of Est1, Est2 (to a lesser extent), and perhaps Yku to TLC1. Thus, unlike the described roles for Pop proteins in tRNA, ribosomal RNA, and mRNA processing, in telomerase, they play a structural role that maintains the integrity of the holoenzyme.

Almost all of the telomere phenotypes in *pop* cells can be explained by the downstream consequences of unstable binding of proteins to TLC1. However, this instability does not provide an obvious explanation for increased TLC1 abundance. To address this question, we collaborated with Harold Kim (Georgia Institute of Technology). His lab used a novel FISH approach to examine the subcellular localization of TLC1 in *pop* and WT cells. In WT cells, TLC1 is transcribed in the nucleus, transits to the nucleolus where it acquires a tri-methylated 5' cap and moves to the cytoplasm where it binds protein subunits to form the holoenzyme. Specific nuclear import factors escort the holoenzyme back to the nucleus where it binds and lengthens telomeres (**Fig. 2a**). By FISH, TLC1 is detected in the nucleoplasm, the nucleolus and the cytoplasm in both WT and *pop* cells. However, compared to WT cells, TLC1 is significantly over-represented in the cytoplasm at 24°C, an effect exacerbated at 30°C. The nucleases that degrade TLC1 are nuclear-localized. Thus, the over-representation of TLC1 in the cytoplasm provides a satisfying explanation for its elevated copy number. We propose that this mis-localization is due to inefficient recognition of the unstable protein-associated TLC1 by nuclear import factors that facilitate nuclear re-entry of the holoenzyme. **Figure 2** presents our view of the impact of Pop proteins on TLC1 biogenesis.

**Figure 2:**
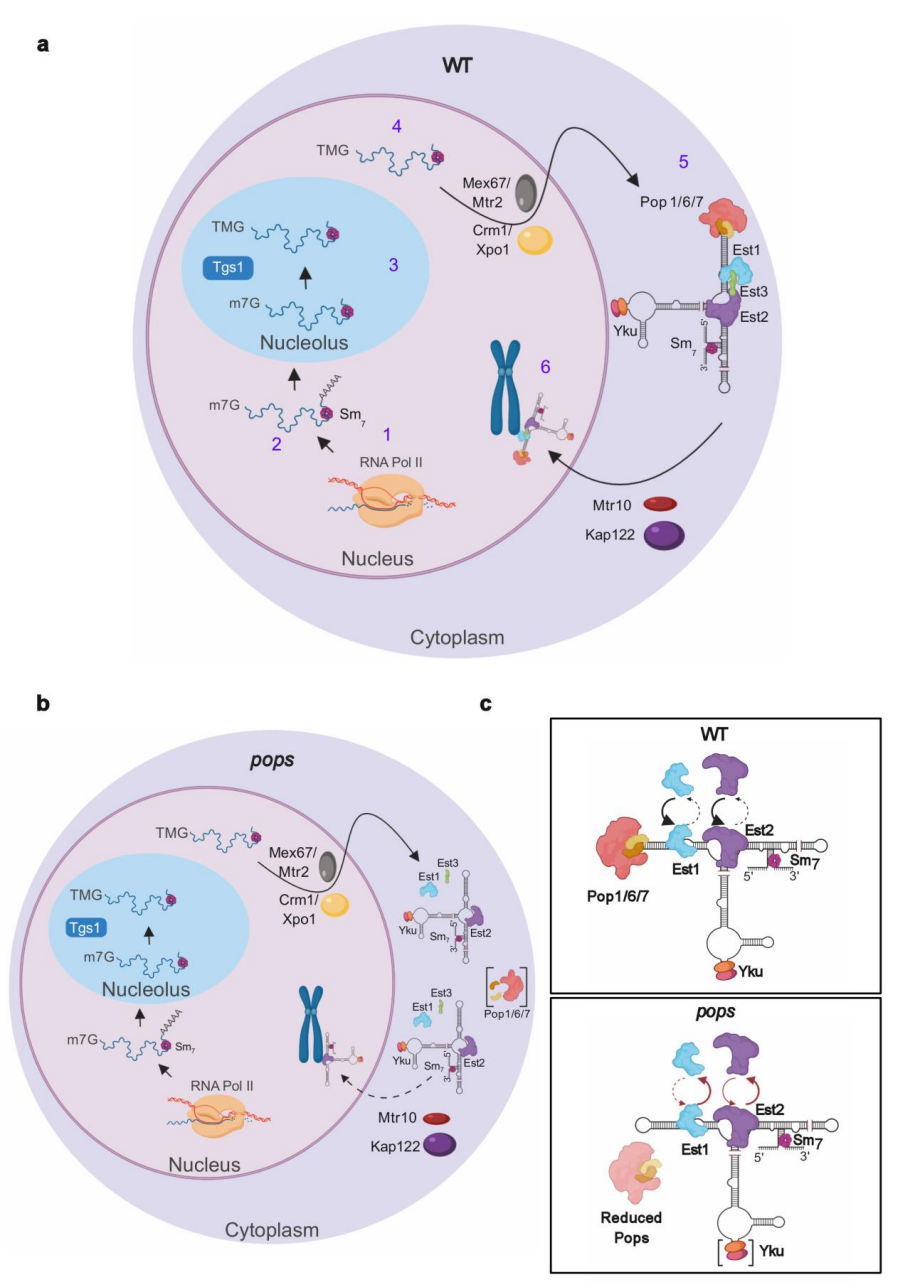
FIGURE 2: The stability and nuclear localization of telomerase is impaired in *pop* cells. **(a)** Steps in TLC1 biogenesis. 1) TLC1 is transcribed by RNA polymerase II with a 5' cap and can acquire a 3' polyadenylated tail. 2) TLC1 is bound by the Sm_7_ proteins that stabilize the RNA. 3) TLC1 is transported to the nucleolus where the 5' cap is hypermethylated by Tgs1. 4) Mature TLC1 lacking a polyadenylated tail is transported to the cytoplasm by nuclear export factors where 5) TLC1 assembles with the protein components to form the telomerase holoenzyme. 6) The mature telomerase is imported into the nucleus by import factors and binds telomeres. **(b)** In *pop* cells where Pop proteins are highly depleted (Pops in brackets), the stability of the Est1 and (to a lesser extent) Est2 binding to TLC1 is reduced leading to an unstable telomerase holoenzyme that is not recognized efficiently by import factors. As a result of this cytoplasmic accumulation, TLC1 is not accessible to the nucleases that degrade it. **(c)** In WT cells (top panel), Pop proteins promote the stable binding of Est1, Est2, and possibly Yku (bottom panel, brackets) to TLC1. In *pop* cells (bottom panel), these proteins still bind TLC1, but this binding is unstable. We propose that the Pop proteins act like chaperones to stabilize the higher-order structure of TLC1, which is needed for the stable association of proteins and formation of the telomerase holoenzyme. Images were made in BioRender (biorender.com).

Is the unanticipated role of Pop proteins in TLC1 biogenesis unique to *S. cerevisiae*? By mass spectrometry, we find that Pop1 co-purifies with fission yeast telomerase so Pop proteins may affect telomerase RNA in other organisms. In addition, there may be other as yet undiscovered RNA-protein complexes that are stabilized by Pop proteins.

